# A New Information System for the Management of Non-Epidemic Veterinary Emergencies

**DOI:** 10.3390/ani10060983

**Published:** 2020-06-05

**Authors:** Luigi Possenti, Lara Savini, Annamaria Conte, Nicola D’Alterio, Maria Luisa Danzetta, Alessio Di Lorenzo, Maria Nardoia, Paolo Migliaccio, Susanna Tora, Paolo Dalla Villa

**Affiliations:** Istituto Zooprofilattico Sperimentale dell’Abruzzo e del Molise “G. Caporale”, Campo Boario, 64100 Teramo, Italy; l.possenti@izs.it (L.P.); l.savini@izs.it (L.S.); a.conte@izs.it (A.C.); n.dalterio@izs.it (N.D.); m.danzetta@izs.it (M.L.D.); a.dilorenzo@izs.it (A.D.L.); maria.nardoia@gmail.com (M.N.); p.migliaccio@izs.it (P.M.); s.tora@izs.it (S.T.)

**Keywords:** non-epidemic emergencies, veterinary emergencies, web GIS, information system, web application, livestock, companion animals

## Abstract

**Simple Summary:**

Veterinary services, along with public health professionals, can be involved in major adverse events resulting from processes of the Earth striking both humans and animals, so-called non-epidemic emergencies. A dedicated information system able to collect specific data and tprovide information to stakeholders is a pre-requisite for the management of these events. The Istituto Zooprofilattico Sperimentale dell’Abruzzo e del Molise “G. Caporale”, home of the National Reference Centre for Urban Hygiene and Non-Epidemic Emergencies (IUVENE), has been entrusted with the development of a system that provides different veterinary operational levels with a spatial and decisional tool to assist them in cases of natural disasters. This kind of decision support system is still missing at a national level. The present paper describes the implementation of the Veterinary Information System for Non-Epidemic Emergencies (SIVENE), an innovative, flexible, and user-friendly system and modular tool including a database, web application, mobile app, and Web Geographic Information Systems (GIS) component. SIVENE provides National Veterinary Services (local health units and national and regional veterinary services) with an emergency management tool responding to the needs identified in the context of catastrophic events, thanks to the data maintained within its database and converted into real time information.

**Abstract:**

The Italian National Veterinary Services, public health professionals, and policy makers are asked to participate at different levels in the decision-making process for the management of non-epidemic emergencies. A decision support system offering the different administrative and operational emergency management levels with a spatial and decisional tool to be used in the case of natural disasters is still missing at the national level. Within this context, the Italian General Directorate for Animal Health of the Ministry of Health funded a research project for the implementation of a new Veterinary Information System for Non-Epidemic Emergencies (SIVENE), an innovative real-time decision support tool for emergency response in a disaster management scenario. SIVENE was developed according to a multi-layer architecture with four integrated components: the database layer, which was implemented by an RDBMS Oracle 11 g; the ReST service layer, which was created using J2EE, Spring, and MyBatis technologies; the web application (business framework and user interface), which was developed in Angular4 framework using TypeScript language; and the web Geographic Information Systems (GIS), which was realized through the implementation of a geodatabase in Oracle RDBMS 11 g. This system allows us to build up and dynamically create a set of dedicated checklists to be used in the field when gathering the information needed for the management of non-epidemic emergencies; employ the application on mobile devices, such as tablets and smartphones; and use the web GIS to manage and visualize data of veterinary interest and territorial maps of risk and damage.

## 1. Introduction

Worldwide natural disasters are growing in frequency, complexity, and severity and are exacerbated by challenges such as climate change [1], complete deforestation [2], and anthropization processes [3].

Natural disasters of different origins can be classified as geophysical, meteorological, hydrological, climatological, biological [4], and extraterrestrial [5].

These major adverse events resulting from the processes of the Earth involve both humans and animals, thus generating so-called non-epidemic emergencies. According to the Centre for Research on the Epidemiology of Disasters (CRED), 1.3 million people have been killed and many others injured, made homeless, or displaced by geophysical and extreme weather events between 1998 and 2017 [6]. The agricultural sector is often heavily affected by these events, with multiple effects on production, food security, and rural livelihoods. The ongoing rising incidence of weather extremes might also have increasingly negative impacts on outbreaks of disease, harvests, livestock, and the destruction of rural infrastructure [7].

Hundreds of thousands of farm animals died in the 2011 flood, which was one of the most catastrophic events that has occurred in Thailand over the last 50 years [8]. Australian estimates suggest that more than 800 million animals have been killed in 2019 bushfires in New South Wales, while international teams provided active response efforts on the ground to rescue and assist koalas, wallabies, flying foxes, birds, and kangaroos. [9].

On 12 March 2020, the New Zealand Government declared a large-scale adverse drought event with consequent pasture cover, stock condition, and feed supplements below average across several regions and unlocked substantial funds to assist primary sector communities and provide farm management advice and animal welfare support [10].

In these critical circumstances, companion animals always represent a source of mayor concern due to the welfare issues involved. Owned dogs and cats might be abandoned or lost and the numerical increase of stray or free-roaming ones pose additional risks to public health and safety, including the potential transmission of zoonotic diseases [11]. These effects may further increase the pressure on animal health infrastructures involved in disaster response [12]. Moreover, emergency management decisions and actions should take into account the link between human well-being and interactions with animals [13,14], and failing to recognize this powerful bond can result in a risk factor for individual and community compliance to evacuation plans [15].

During these large-scale disasters, information about affected people, infrastructure, and resources need to be urgently handled by multiple organizations that produce and share considerable amounts of data. However, the majority of information are still provided as written reports, documents, or tables in various formats. Consequently, a relevant amount of time is needed to collect and combine them into a comprehensive situational overview, allowing all actors involved in the emergency management system to meet their needs and responsibilities [16,17].

In light of this, both in peace time and during the management of an adverse event, a dedicated and unique information system able to collect specific data and provide information to stakeholders is a pre-requisite for the management of these events. This kind of decision support system for non-epidemic veterinary emergencies is still missing at the national level.

The need for an information system has emerged during the last decade also in the field of veterinary medicine. In fact, in the event of natural disasters, veterinary expertise is often called upon by other authorities and animal keepers. In particular, veterinary services are directly involved in the response and recovery phases whenever farm and companion animals’ health and welfare, food and feed safety, and security are at risk. Other important aspects beyond acute animal welfare issues are also under their competency, like food and feed safety, veterinary public health, pest control an disinfection, and the disposal of carcasses.

In recent years, moreover, several spatial decision support systems have been used by a huge portion of veterinary science for animal disease surveillance and monitoring at national and international levels. Geographic Information Systems (GIS) have been used for understanding the dynamics and spatial patterns of a disease, performing cluster analyses, generating and testing hypotheses of disease causation, the management of epidemic emergencies, and planning control strategies [18,19]. GIS has been widely applied to different veterinary purposes for producing maps of disease incidence, prevalence, mortality, and morbidity in a specific geographical area or at the farm level. Furthermore, in case of an outbreak of infectious disease or during epidemic emergencies, it is an excellent tool for identifying the location of the case farm and all the farms at risk within a specified buffer around the outbreak [18].

Furthermore, the wide availability of information that public veterinary institutions and organizations are called to collect at a national and international level through the World Organisation for Animal Health (OIE) Information System WAHIS, the World Health Organization (WHO), and the Animal Disease Notification System (ADNS–European Commission) have been a valuable source of information for the management of epidemic emergencies.

Since 2009, the OIE has taken a leadership role in identifying the current state of disaster management and risk reduction processes in order to enhance veterinary services’ resilience and capacity to manage non-epidemic emergencies. Within this context, the OIE recommended the implementation of a specific conceptual framework covering all phases of the Disaster Management Cycle (prevention, preparedness, response, and recovery) to be applied in conjunction with existing international, regional, and national instruments [20]. Over the last two decades, the Food and Agriculture Organization of the United Nations (FAO) has also seen a major increase in requests for assistance in responding to disasters, and it has been promoting the development of guidelines and standards focusing on the management of livestock emergencies [21,22,23].

To date, the increasing computational power of modern technologies, together with advanced informatics tools, have enabled the collection and sharing of standardized data [21,22,23,24]. In particular, GIS, remote sensing, and the Internet had a significant impact and are currently used in several ways during all phases of the disaster management cycle [25]. Integrating these technologies to create a spatial decision support system offers new possibilities for disaster management, particularly during the initial response phase when the ability to detect changes is more urgent and access to relevant information and spatial data are among the most essential requirements [26,27,28]. GIS synthesizes information from a vast number of different data sources and helps in assessing the impacts of disaster and planning response and relief strategies.

Today, several GIS-based solutions are already available on the web, aiming at improving emergency management and increasing the connectivity and data sharing between local, national, and international organizations (e.g., Global Earth Observation System of Systems (GEOSS) [29]; Copernicus Emergency Mapping Services (EMS) [30]; etc.).

In parallel, the implementation of specific tools complementing the ones already existing for the management of veterinary emergencies at the national level is still missing. This tool would enable one to estimate farms’ and animals’ vulnerability and the size of the impacted areas. This would facilitate the preparation and implementation of emergency plans, both during the definition and the allocation of logistic support (feed, medical care, evacuation, etc.) as well as during disaster damage and loss assessment interventions.

Since 1992, the Italian Veterinary Services, along with the other components of the National Health Services, are fully integrated with the National Civil Protection system at the national, regional, and local level. According to the recently enforced Code of Civil Protection (Legislative Decree 1/2018), this system “*exercises the civil protection function, consisting of all the competences and activities that are designed to protect the life, the physical integrity, the goods, the settlements, the animals and the environment from damage or danger of damage arising from disastrous events of natural origin or arising from human activity*” [31].

Other veterinary public health institutes, such as the Istituti Zooprofilattici Sperimentali-National Reference Centers, which form a network of public laboratories at the national and regional level, operate in the Italian Civil Protection system as part of the “support function” F2-Health, social, and veterinary assistance [32].

During the past, catastrophic events have occurred in central Italy (e.g., the earthquakes of Aquila 2009, Amatrice 2016, and Norcia 2017) the National Reference Centre for Urban Hygiene and Non-Epidemic Emergencies (IUVENE) Information System, already used as a prototype in support to the veterinary actions conducted in the aftermath of the L’Aquila earthquake in 2009, was made available again to the Veterinary Services by the the “Istituto Zooprofilattico Sperimentale dell’Abruzzo e del Molise G. Caporale (Teramo, Italy)”.

This decision support system has proven to be a fundamental operational tool to support a coherent and harmonized data collection on the main criticalities notified at the farms, food establishments, feed industries, dog shelters, and pet facilities level. On this occasion, the system was updated and adapted to better plan and prioritize veterinary interventions needed for the assistance of companion animals temporarily displaced with their owners, lost, free roaming, or stray [33].

Later in 2017, in the aftermath of other seismic sequences and heavy snowstorms that hit the Abruzzo region again, this information system was further updated in order to increase the efficiency of the rescue and recovery actions [34]. At that time, the system already allowed the user to verify and monitor the effectiveness of the different actions undertaken and rapidly put in place proper corrective measures.

The difficulties experienced in these circumstances clearly highlighted the need for a new system design that is technologically more advanced, more flexible, more user-friendly, and able to manage different kinds of hazards over the entire national territory. To achieve this purpose, the Italian General Directorate for Animal Health and Veterinary Medicinal Products of the Ministry of Health funded a research project for the implementation of a new Veterinary Information System for Non-Epidemic Emergencies (SIVENE), an innovative web application able to respond to specific identified needs in the context of non-epidemic emergencies. The new information system allows users to manage, display, and analyze data and information generated by the Veterinary Services involved in the response and recovery phases. The results obtained and the new knowledge generated by this study are the basis for the correct implementation of monitoring systems for non-epidemic veterinary emergencies.

## 2. Materials and Methods

For SIVENE’s development, specific attention was paid to some features that are extremely useful in the management of veterinary emergencies, in particular:

- The possibility of using the application on mobile devices (tablets and smartphones) regardless of the platform;
- The possibility to dynamically generate new standardized checklists for data collection and analysis during situational awareness in-field inspections carried out by veterinary rescue teams;
- A natural integration with the web GIS.

## 3. System Development and Architecture

**Figure 1:**
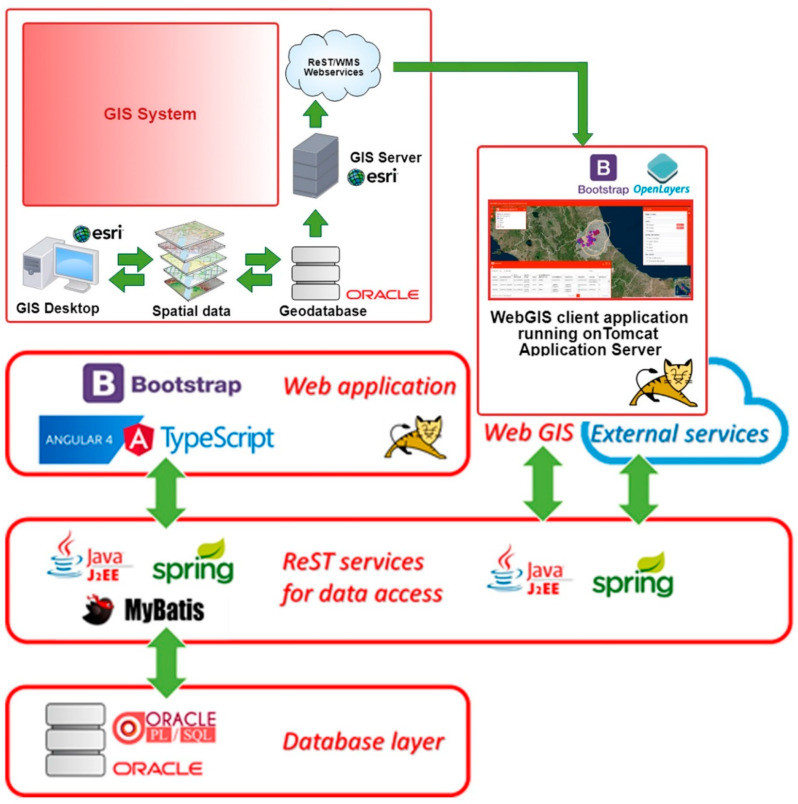
SIVENE architecture.

**Figure 2:**
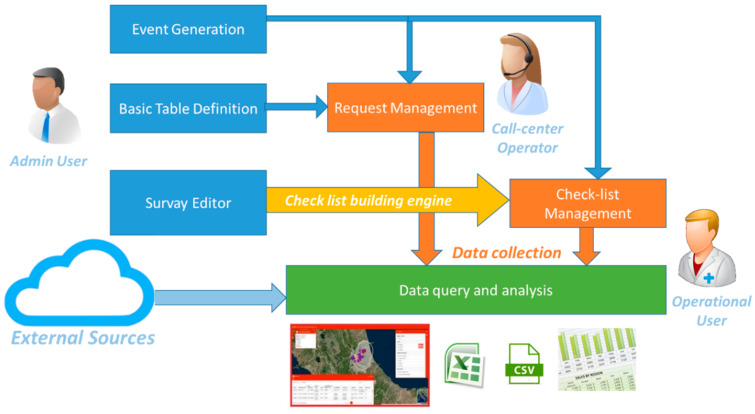
Veterinary Information System for Non-Epidemic Emergencies (SIVENE) software workflow chart. The principal application features are organized in a software workflow chart with indication of different type of users.

The new information system was developed according to a multilevel (or multi-layer) architecture, as shown in Figure 1. This architecture allows greater flexibility and independence in the software implementation and the possibility of decoupling various components of the application itself. In particular, four components were implemented and integrated: the database layer, the ReST service layer, the web application, and the web GIS. Figure 2 shows an overall system framework and the principal SIVENE features organized in a software workflow chart in order to highlight the emergency management through the provided tools: definition of new emergency events; decoding and work tables; management of requests for assistance; survey editor and dynamic check-lists; check-list management and data collection; data query and analysis.

The database layer allows the recording and storage of data, and it is implemented by an RDBMS Oracle 11 g. It consists of structured tables for storing information and an application layer, developed with PL/SQL procedures, to process and standardize the data. The use of an RDBMS ensures easy and efficient access to data, maintaining its consistency, privacy, and reliability.

The rest service layer was created using the J2EE, Spring, and MyBatis technologies to connect with the database and the application user interface. This layer provides a set of services related to all the functionalities of the system and also enables the total decoupling between the data and application interfaces. Thanks to these services, it is possible to develop, at the front-end level, a web application rather than other external desktop or mobile applications. This layer also ensures communication and interfacing with the Geographic Information Systems (GIS) system.

The web application is the most consistent part of the entire architecture. It was developed in Angular4 framework using TypeScript language and includes both the final graphic interface and the business logic. Following the guidelines of the Italian Agency for Digitalization (AGID) and using the Bootstrap CSS framework, a modern graphic interface was developed, also creating well-defined software components as data tables, list of values, etc. All the graphic components and related styles are standardized, defined, and developed during the project and collected in an external software library included into the application but also available and reusable for other projects.

**Figure 3:**
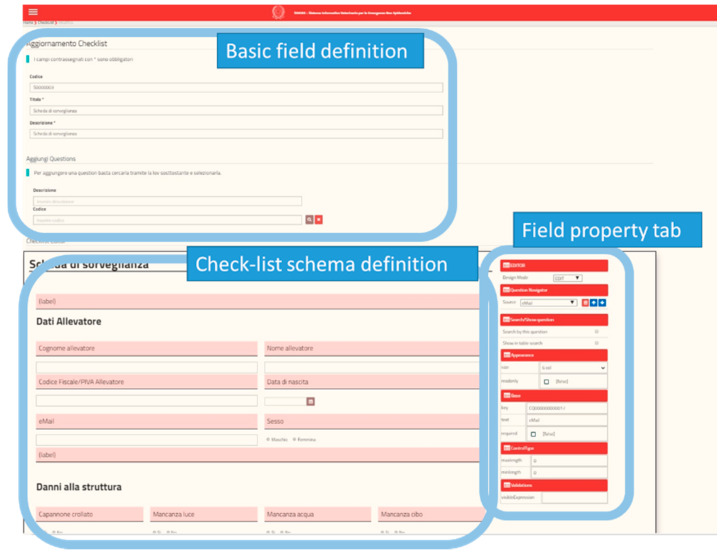
Survey editor to dynamically build checklists. The admin user, using this feature, can build a new checklist. The figure shows the different sections of the tool: a section for a basic element definition; a section to define the schema of a new checklist where, using drag-and-drop, it is possible to combine several basic elements; and a property tab to define the basic characteristics of each field (such as type, position, dimension, etc.).

**Figure 4:**
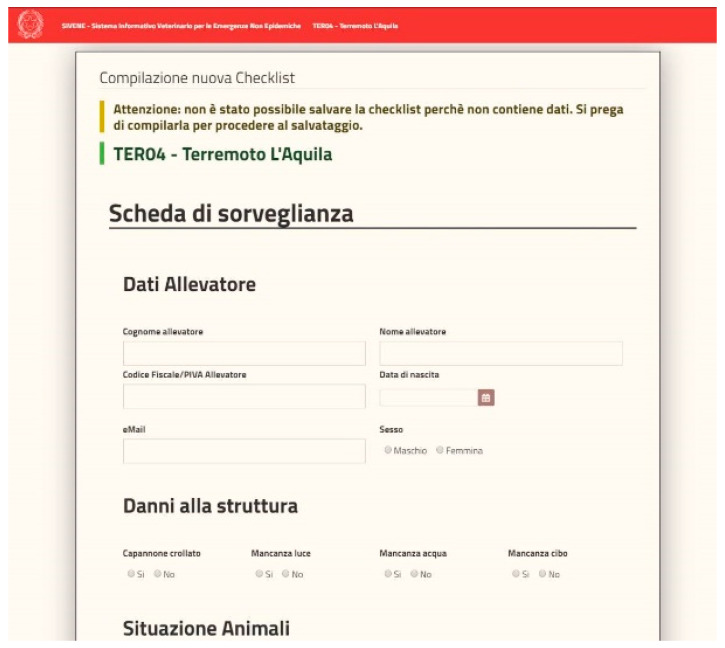
Web form example of the dynamic checklists used for the entry of damage data collected for the planning of intervention measures (i.e., farmer’s personal information, type of structural damages, and number of dead or injured animals).

From the application point of view, the SIVENE system is composed of a public and a private area. The public area, freely available without authentication, can be useful for the publication and dissemination of news and information relating to the emergency. The private section allows only authorized users, each one with their own role, to access the operational functions. The principal features of the private area of SIVENE web application are:

- The definition of new emergency events: among the functionalities available in SIVENE, there is the possibility to define and characterize, even geographically, an event, its source, and the area of interest in a simple way through simple functionalities provided by application forms.
- Decoding and worktables: SIVENE allows the user to manage the decoding and basic tables such as the definition of an event or a type of request.
- The management of requests for assistance: a specific functionality of the system allows registering all requests coming from the field collected (for example, by a specific dedicated call-center) during the emergency. The requests can be classified and displayed, also on the maps, by the users to identify the main problems and properly target the actions.
- Survey editor and dynamic checklists (Figure 3): the SIVENE system includes an ad hoc engine to create dedicated checklists and deliver them. To create these checklists (or surveys), it is necessary to define firstly the basic fields, called individual questions, that represent the basic elements of a survey, such as, for example, a text box for a name, a surname, or an email rather than a date-field or a table of data for more complex fields. The question editor is the basic module of the engine in which the individual questions are conceived to be composed in order to build the survey. The system proposes a list of basic individual questions that can be used or customized for the development of dynamic checklists. The survey editor allows an administrative user to define the different fields (of different data types) and include them in a new specific checklist according to needs.
- Checklist management and data collection: the system presents to the operational users different checklists, based on this dynamic definition, as different forms (Figure 4) for data collection. These forms can be printed and the data collected on paper or filled (directly from the field or after data collection on paper) using a web application from a PC or mobile devices (tablet or smartphone).
- Data query and analysis: data stored and managed by SIVENE are related to the information concerning requests for assistance or collected during in-field inspections by using dedicated check-lists. The data could include the magnitude and the geographical coordinates of the epicenter of the earthquake (data source: National Civil Protection system); the address and geographical coordinates; and the type of livestock holdings and farms (i.e., slaughterhouses, staging points, animal markets, breeding, and assembly centers), the type of animal production (i.e., meat, eggs, milk, and wool), the number of animals, and the species held in the farms (data source: National Data Bank) affected by the disaster. SIVENE users can extract all the data collected in different formats as raw data in csv, tab delimited, xls, etc. or as preconstructed data tables, graphs, maps, etc. for further analysis.

The new information system was also equipped with an ad hoc engine for the dynamic creation of checklists, which consist of forms for the collection of specific information on the control and inspection activities carried out by the operators in case of an emergency.

### GIS System

The geospatial analysis and visualization play a fundamental role in the early warning and post-event emergency management. Consequently, basic spatial information and thematic maps are stored and managed in a geospatial database so as to display, analyze, and query the data through a map viewer. Figure 1 shows the GIS system architecture schema integrating Geodatabase, GIS, and web GIS. The first web GIS application is dedicated to a seismic emergency.

The Geodatabase was designed to manage and integrate geospatial data provided as input to the new information system, including the alphanumeric data related to:

1. Emergency events: magnitude and epicenter of the event, the seismic hazard map recorded and processed by the CPD, and the estimated seismic livestock vulnerability.
2. Exposed elements: holdings, livestock farms, and their relevant attributes (i.e., livestock farming types: slaughterhouses; staging points; animal markets; assembly centers; holding type; production of meat, eggs, milk, and wool; address and geographical coordinates of the holding; number of animals per holding; animal species) extracted from the NDB.
3. Basic geographic layers: regions; municipalities; and street, topographic, simplified, and satellite maps as Tiled Map Services by ESRI^®^.

The technology used to implement the Geodatabase was Oracle RDBMS 11 g.

**Figure 5:**
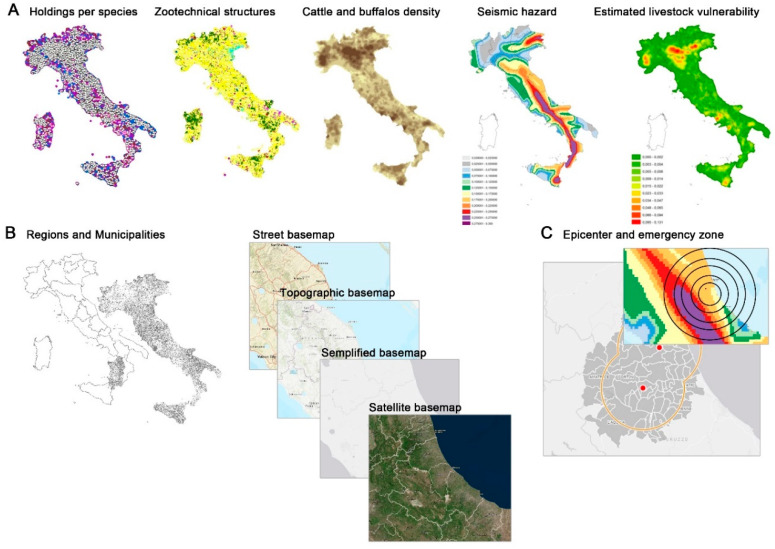
(**A**) Geodatabase datasets: holdings per species, livestock farms, animal density per species, the seismic hazard, and the estimated seismic livestock vulnerability. (**B**) Additional layers displayed in the web GIS: administrative boundaries (regions and municipalities) and base maps, including street, topographic, simplified, and satellite. (**C**) The simulated seismic zone.

The GIS meanly deals with the processing of geographical data and spatial information. Spatial analysis procedures and geo-processing operations provide a complete and update description of the study area and, as final result, a map of estimated seismic livestock vulnerability and the animal density per species with a spatial resolution of 1 km (a kernel density function with a radius of 15 km has been used for data processing [35]. The software package used to implement the GIS section was ESRI^®^ ArcGIS Desktop 10.5. The web GIS application was then designed, mixing open source and proprietary technologies in a client-server architecture, providing maximum flexibility and the possibility to easily integrate spatial data coming from different sources and formats. The server-side component consists of an ESRI ArcGIS Server connected to an Oracle RDBMS to expose the spatial data as a series of ReST map services. The map services are queried by a JavaScript client realized with the open source library OpenLayers to develop the GIS functions and the Bootstrap CSS Framework to create custom UI widgets. The web GIS application was deployed in a J2EE Environment using the Tomcat Application Server. The datasets collected in the Geodatabase and displayed by the web GIS are summarized in Figure 5.

## 4. Results

SIVENE is a useful and innovative tool for data management during veterinary non-epidemic emergencies. It is technologically advanced, both in terms of architecture and in terms of advanced features; flexible; and user-friendly. The system is a research product owned by the Ministry of Health, and its software code is currently not publicly available as an open source project.

The SIVENE web application allows the user to dynamically create dedicated checklists able to gather the information needed during an emergency. The Veterinary Services or other competent authorities can collect (both in online or offline modes) data regarding the status of buildings, facilities, animal health status, and supporting infrastructure during the inspection of the stables or other relevant structures. The collection of these data is allowed by an ad hoc survey form provided by the application. Therefore, the data collected can be used to create an immediate picture of the situation that can be displayed on maps for further consideration and analyses.

**Figure 6:**
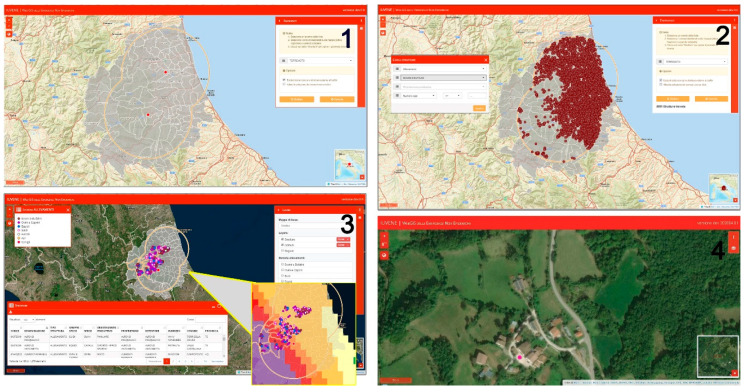
Web GIS application workflow: (**1**) Identification of municipalities involved in the emergency area. The map shows the earthquake epicenters (red dots) and the administrative units involved (selected by user through the “search” toolbox, in the upper right, listing all emergency events stored in the system) in the emergency area of interest. (**2**) The “exposed elements” (livestock farms) falling in the emergency area can be identified and showed on the map as dots of different colors using the “data filter” tab based on a predetermined list of different types of structures, animal species, and type of productions (meat, eggs, milk, and wool). (**3**) The “data filter” tab allows the user to refine the search for holdings selected according to specific criteria (e.g., number of animals> 100), and a set of related data is shown on the map. The legend (per the holding layer) at the top left helps reading the map; the dots are colored according to the species; and the “table” tab at the bottom right, showing the information related to each farm (i.e., address, owner, species, number of animals, production type, etc.), is exportable as an excel file. The farms insisting on a specific risk level can be easily displayed by selecting the seismic hazard layer on the tab panel at the top right. (**4**) A zoomed farm (fuchsia dot) on the satellite map also allows the user to easily visualize details of the farm position and accessibility.

The key features and strengths of the system are:

- Interface and user management: a new interface has been designed to be usable natively by mobile devices and according to the guidelines suggested by AGID (the Italian Agency for Digitization) for Public Administration web applications.
- Integration with the Italian National Animal Identification and Registration System and the Livestock Data Bank (NDB): the integration with the NDB also allows users to connect in real time and in relation to the specific event the data of the livestock or the list of the farms and related animals present in the area affected by the emergency.
- Survey editor and dynamic checklists: the system was also equipped with an ad hoc engine for the dynamic creation of checklists. Structural damage and the interruption of services and roads as well as animal health and welfare data can be collected during in-field inspections carried out by veterinary rescue teams. The survey editor ensures the system a high flexibility and the possibility to customize data collection, based on the different needs that each new emergency may present.
- Web GIS: the SIVENE web GIS is organized in a map window showing the spatial data on a base map and a series of floating panels used to easily select the event of interest by a dropdown list, in order to build the queries to turn on and off informative layers and to visualize information associated with the spatial data as downloadable attribute tables for farms involved in the emergencies. Figure 6 show a simulated operations flow to improve knowledge, at the local level, of the veterinary sites of interest involved in the emergency event, by using the SIVENE web GIS tools.

## 5. Conclusions

The nature and complexity of non-epidemic veterinary emergency management have grown significantly over the past decade. However, there is still a need for decision support systems that can be used for emergency planning, response, and recovery and to facilitate decision-making when veterinary services are involved in crisis situations. In the context of a recent earthquake that occurred in Italy, the National Reference Centre for Urban Hygiene and Non-Epidemic Emergencies (IUVENE) supported the activities of the national, regional, and local veterinary services operating within the Civil Protection Function F2, “Public health, social and veterinary assistance”, through the IUVENE information system employment.

In light of this experience, and thanks to the support of the Italian Ministry of Health, an innovative Veterinary Information System for Non-Epidemic Emergencies (SIVENE) was developed as part of the research project, “Management of veterinary activities during non-epidemic emergencies”. The SIVENE allowed the management and displaying of all the data collected by various end-users and the assessment of the information generated during the event, as well as disseminating updated real-time data in an efficient way. Therefore, this system was an essential tool to support the activities of veterinary services during the emergency response and recovery phases. The system is naturally integrated with the Italian Animal Identification and Registration system and other Italian national registers. Thanks to an extremely flexible rest services layer, it is adaptable to different contexts by upgrading its level of interoperability with other countries’ existing registers and databases. The system language is currently only in Italian, but its architecture allows it to be easily translated into other languages.

The web GIS application is the best way to show and share georeferenced information about the emergency on the web. Nowadays, web GIS application sand geospatial web services are often used as decision support systems both in public and animal health fields. GIS tools make it possible to immediately determine the location of farms and zoo technical structures, both in absolute terms and in relation to an area or a point of interest. This knowledge is critical and helps to establish the priority for interventions during an emergency. Having such tools in a web application makes all the process easier, faster, and accessible also for users that are not GIS experts.

These tools were applied to analyze the potential hazard that would result from a defined magnitude earthquake. A first study was conducted to develop a procedure for mapping and assessing estimated seismic livestock vulnerability, integrating spatial and geoprocessing tools. In particular, an estimated seismic livestock vulnerability map was produced using the position of cattle farms, the number of cattle, and the local seismic intensity values. To date, SIVENE functions have been presented in the frameworks generated by the “North-Eastern International Flood Exercise (NEIFLEX)” held in Veneto in 2018 and made available to the regional and local veterinary services involved in the “GIOTTO 2019” seismic emergency simulation exercise held in Tuscany in 2019. On these occasions, the IUVENE National Reference Center, in collaboration with the Statistic and GIS Department and the National Service Center for Animal Registration, had the opportunity to present, test, and validate the SIVENE key features in the context of Civil Protection drills. Thirty-two requests for assistance were simulated for the “GIOTTO 2019” exercise and three specific checklists generated by SIVENE were made available to the rescue teams engaged in preliminary inspections at farm, food companies, and private and public dog shelters levels. The data collected in-field were managed in real time, and decision-makers had the option to verify the outcomes of each inspection in order quickly prioritize the planned interventions. Furthermore, during the debriefing phase, an analysis of SIVENE’s strengths and weaknesses was carried out for further emergency use. This system is currently addressed to local, regional, national Veterinary Services and Civil Protection users, though its access could be further extended to other stakeholders.

In the near future, SIVENE will be included in an Information Platform able to define both the vulnerability and damage scenarios according to different types of risks. In addition, the Sentinel images of the Copernicus mission [35], which have a global coverage with high spatial resolution and time interval of 5 days, could be also integrated. The acquisition of these satellite images before and after the non-epidemic event would allow a more detailed analysis in the affected areas. Specific indicators on the vulnerability of livestock, farms, food, and feed establishments will be also included in the model (e.g., elevation index, Equivalent Bovine units values (EBU), the distance from main roads, etc.) in order to have a more realistic result.

To date, SIVENE already represents one of the most advanced tools to be used for strengthening and optimizing preparedness and response veterinary actions during the management of non-epidemic emergencies. However, progressive technological improvements would be of great significance for the development and implementation of Veterinary Service’s disaster management and risk reduction plans in different scenarios, understanding that the future may hold other new and unexpected threats to humans, animals, and the environment.
